# Novel Therapeutic Targets of Endothelial Inflammation in Acute Lung Injury and Acute Respiratory Distress Syndrome

**DOI:** 10.70322/jrbtm.2026.10001

**Published:** 2026-02-06

**Authors:** Yunchao Su

**Affiliations:** 1 Department of Pharmacology & Toxicology, Medical College of Georgia, Augusta University, 1120 15th Street, Augusta, GA 30912, USA; 2 Division of Pulmonary and Critical Care Medicine, Department of Medicine, Medical College of Georgia, Augusta University, Augusta, GA 30912, USA; 3 Vascular Biology Center, Medical College of Georgia, Augusta University, Augusta, GA 30912, USA; 4 Research Service, Charlie Norwood Veterans Affairs Medical Center, Augusta, GA 30912, USA

**Keywords:** Lung, Endothelium, Inflammation, Barrier function, Acute lung injury, Acute respiratory distress syndrome

## Abstract

Lung microvascular endothelial inflammation and barrier dysfunction play critical roles in the pathogenesis of acute lung injury (ALI)/acute respiratory distress syndrome (ARDS). Despite recent scientific advances, the mortality of ALI/ARDS is still extremely high because the molecular mechanisms involved in ALI/ARDS remain unclear. In a recent issue of the journal Advanced Science, Baoyinna and colleagues reported that deubiquitinase USP30 induces lung microvascular inflammation and endothelial barrier disruption through the S-adenosylmethionine (SAM) cycle, DNA methylation, and miR-30a-5p down-regulation in ALI/ARDS. Their findings provide a strong rationale for targeting microRNAs, S-adenosylmethionine, DNA methylation, and deubiquitinating enzymes as potential therapeutic strategies for the treatment of ALI/ARDS.

Acute lung injury (ALI)/acute respiratory distress syndrome (ARDS), characterized by diffuse lung microvascular inflammation, endothelial dysfunction and pulmonary edema, is a clinical syndrome caused by severe organ injury, infection or sepsis [1,2]. Despite recent scientific advances, the mortality of ALI/ARDS is still extremely high because molecular mechanisms involved in ALI/ARDS remain obscure. Therefore, there is an urgent need to identify potential therapeutic targets for the treatment of ALI/ARDS.

In a recent issue of the journal Advanced Science, Baoyinna et al. [3] reported that deubiquitinase USP30 is a critical regulator of lung microvascular inflammation and endothelial barrier integrity in ALI/ARDS (Figure 1). They found that endothelial-specific USP30-deficient mice exhibit reduced lung endothelial inflammation and microvascular barrier dysfunction in models of endotoxin-induced and ischemia-reperfusion lung injury. They discovered a novel mechanism by which endotoxin induces inflammatory responses in ALI/ARDS. The data revealed that USP30 deubiquitinates and stabilizes methionine adenosyltransferase 2A (MAT2A), leading to increased S-adenosylmethionine (SAM) and DNA methylation, and reduced miR-30a-5p expression in ALI/ARDS. Their results further indicate that miR-30a-5p suppresses the expression of mouse double minute 2 homolog (MDM2) and nuclear factor of activated T cells 5 (NFAT5), thereby alleviating endothelial inflammation and protecting endothelial barrier function. Their findings provide a strong rationale for targeting microRNAs, S-adenosylmethionine, DNA methylation and deubiquitinating enzymes for potential therapeutic strategies in ALI/ARDS (Figure 1).

## 1. MicroRNAs

MicroRNAs target specific mRNAs to regulate gene expression, either by blocking translation or promoting mRNA degradation in cells, thereby influencing endothelial cell homeostasis by modulating processes such as cell monolayer permeability, cell apoptosis, and the inflammatory response in the pathogenesis of ALI/ARDS [4]. miRNAs are significantly upregulated or downregulated in lung endothelial cells or other lung cells and play injurious or protective roles in lung endothelial inflammation [5]. For example, miR-34a-5p, miR-1246 and miR-92a are upregulated in endotoxin-treated pulmonary microvascular endothelial cells and promote endothelial damage and inflammation in ALI [6-8]. miR-34a-5p has been found to target histone deacetylase 1 (*Sirt1*) gene [6], miR-1246 to target the angiotensin-converting enzyme 2 (*Ace2*) gene [7], and miR-92a to target integrin a5 (*Itga5*) gene [8]. These microRNAs could be potential therapeutic targets and diagnostic biomarkers of ALI/ARDS [9-11]. miR-339-3p, miR-539-5p and miR-33 are down-regulated in endotoxin-stimulated lung microvascular endothelial cells and play a protective role in endothelial inflammation in ALI [12]. miR-339-3p was confirmed to target *annexin A3* gene [12,13], miR-539-5p to target Rho kinase (*Rock*) gene [13], miR-33 to target receptor interacting protein 140 (*Rip140*) gene [14]. Over-expressions of miR-339-3p, miR-539-5p and miR-33 alleviates lung endothelial dysfunction, inflammation, and vascular injury in ALI/ARDS. In the study of Baoyinna et al. [3], they reported that miR-30a-5p overexpression downregulated the expression of co-transcriptional factor NFAT5 and MDM2 and inhibited endotoxin-induced expression of vascular cell adhesion molecule 1 (VCAM1) and intercellular adhesion molecule 1 (ICAM1) in pulmonary microvascular endothelial cells, suggesting that miR-30a-5p is a protective microRNA that alleviates endothelial inflammation and barrier dysfunction. Together with miR-339-3p, miR-539-5p and miR-33, these protective microRNAs could be used as novel pharmacologic agents in ALI/ARDS.

## 2. S-Adenosylmethionine and DNA Methylation

DNA methylation is an epigenetic regulatory mechanism that involves the transfer of a methyl group to the C5 position of cytosine at CpG dinucleotide sites, forming 5-methylcytosine in the mammalian genome. Genomic DNA methylation is a dynamic process of methylation and demethylation through DNA methyltransferases (DNMTs) and demethylases. The primary methyl donor for DNA methylation is S-adenosylmethionine (SAM), a species generated in the methionine cycle of one-carbon metabolism [15]. In ALI/ARDS, DNA methylation suppresses gene expression, resulting in pathophysiologic changes in endothelial inflammation and permeability [16,17]. Alterations or manipulations of DNMTs and demethylases would influence inflammatory response and lung vascular barrier function [18-20]. Another mechanism for the regulation of DNA methylation is through the modification of the level of methyl donor SAM [21]. Baoyinna and colleagues found that the reduction of intracellular SAM level caused by MAT2A degradation (inhibition) mitigates DNA methylation of the gene for miR-30a-5p, which leads to down-regulation of inflammation and endothelial permeability [3]. Targeting the SAM cycle and DNA methylation could be a novel therapeutic strategy for acute inflammatory diseases. Interestingly, SAM is also the methyl donor for histone methyltransferase [22], and the SAM level has an impact on histone methylation and gene expression in lung inflammation [23]. Baoyinna and colleagues did not study whether the reduction of intracellular SAM level caused by MAT2A degradation (inhibition) mitigates histone methylation in ALI/ARDS. Further studies are needed to disclose more epigenetic mechanisms of lung inflammation and endothelial barrier regulation.

## 3. E3 Ubiquitin Ligases and Deubiquitinating Enzymes

Protein ubiquitination links ubiquitin to target proteins via E3 ubiquitin ligases, leading to protein degradation via the ubiquitin-proteasome system. Deubiquitinating enzymes (DUBs) can reverse the ubiquitination process by removing the ubiquitin chain from the target protein. The equilibrium between ubiquitination and deubiquitination is essential for maintaining intracellular protein homeostasis and signaling [24]. Ubiquitination and deubiquitination can directly modulate the lung endothelial barrier by controlling the stability and expression of proteins in adherens junction (AJ) and tight junction (TJ), as well as small Rho GTPases that adjust the actin cytoskeleton in lung microvascular endothelium in ALI/ARDS [25-27]. Hakai, an E3 ubiquitin ligase, mediates E-cadherin ubiquitination and degradation [28]. β-Catenin is ubiquitinated by the E3 ubiquitin ligase β-transducin repeat-containing protein (β-TrCP) and then degraded by the proteasome [29]. Itch, a HECT domain-containing E3 ubiquitin ligase, interacts and ubiquitinates occludin in the tight junction, resulting in occludin degradation by the proteasome [30]. Several E3 ubiquitin ligases, such as Smurf1, Cullin3/BACURD, Fbxl19, and Fbxw7, have been identified as regulators of RhoA ubiquitination and stability [31]. Two DUBs, OTUB1 and USP17, have been shown to regulate RhoA ubiquitination and degradation [25].

Ubiquitination and deubiquitination regulate the NLRP3 inflammasome and NF-κB (nuclear factor kappa-light-chain-enhancer of activated B cells) activation in lung inflammation in ALI/ARDS [32,33]. E3 ubiquitin ligases target NLRP3 proteins or their constituents to degrade NLRP3 inflammasomes. In endotoxin-induced ALI model, the expression of E3 ubiquitin ligases Pellino2, WW domain-containing E3 ubiquitin ligase protein 1 (WWP1) and E3 ubiquitin ligase adaptor BTB/POZ domain-containing protein 2 (BPOZ-2) was found to be downregulated [34-36]. Pellino2, WWP1 and BPOZ-2 promote ubiquitination of NLRP3 inflammasomes, thereby downregulating the expression of NLRP3 inflammasomes, IL-1β and TNF-α proteins [34-36]. In ALI/ARDS, YAP, a transcriptional co-activator, interacts with the E3 ubiquitin ligase TRAF6 (tumor necrosis factor receptor-associated factor 6) to promote its degradation via K48-linked ubiquitination and to inhibit K63-linked autoubiquitination in ECs, thereby inhibiting NF-κB activation [37].

Different DUBs act on their specific target proteins and exert different roles in lung inflammation [33]. Several DUBs such as BRCC3, ABRO1, USP7, and USP47 have been shown to be implicated in NLRP3 inflammasome activation [38]. USP9X was found to be upregulated and participate in the pathogenesis of ALI by promoting NLRP3 inflammasome deubiquitination and activation [39]. USP14 protein is upregulated, which reduces I-κB protein levels and thus increases cytokine release in lung inflammation [40]. On the other hand, USP7 acts as a negative regulator of the NF-κB pathway by mediating the deubiquitination of NEMO, TRAF6, and IKK, thereby retaining NF-κB in the cytosol and suppressing its activity and the expression of inflammatory cytokines [41]. In the study by Baoyinna et al. [3], USP30 was inhibited using siRNA *in vitro* and endothelial-specific gene knockout *in vivo*. Inhibition of USP30 reduced MAT2A ubiquitination and degradation, DNA methylation, and increased miR-30a-5p expression, thereby ameliorating vascular leakage, cytokine production, and lung inflammation in ALI/ARDS. USP30 may represent a potential therapeutic target warranting further preclinical and clinical trials in ALI/ARDS.

## Figures and Tables

**Figure 1.**
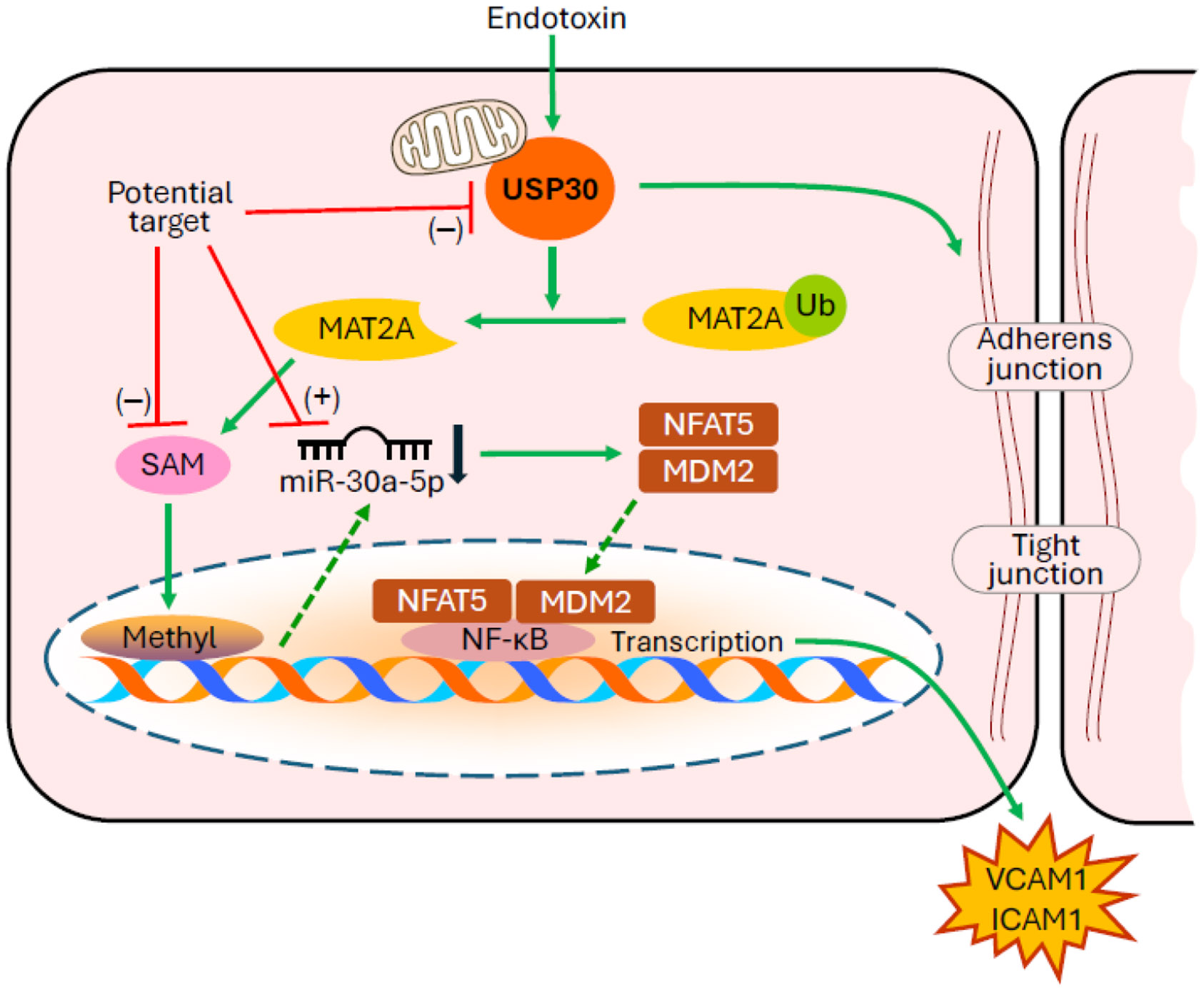
MicroRNAs, S-adenosylmethionine, DNA methylation, and deubiquitinating enzymes are potential targets of endothelial inflammation for therapeutic strategies in the treatment of ALI/ARDS. USP30, ubiquitin-specific peptidase 30; MAT2A, methionine adenosyltransferase 2A; SAM, S-adenosylmethionine; NFAT5, nuclear factor of activated T cells 5; MDM2, mouse double minute 2 homolog; NF-κB, nuclear factor kappa-light-chain-enhancer of activated B cells; VCAM1, vascular cell adhesion molecule 1; ICAM1, intercellular adhesion molecule 1.
